# Dynamic responses of DOC and DIC transport to different flow regimes in a subtropical small mountainous river

**DOI:** 10.5194/hess-22-6579-2018

**Published:** 2018-12-21

**Authors:** Yu-Ting Shih, Pei-Hao Chen, Li-Chin Lee, Chien-Sen Liao, Shih-Hao Jien, Fuh-Kwo Shiah, Tsung-Yu Lee, Thomas Hein, Franz Zehetner, Chung-Te Chang, Jr-Chuan Huang

**Affiliations:** 1 Department of Geography, National Taiwan University, Taipei, Taiwan; 2 Department of Civil and Ecological Engineering, I-Shou University, Kaohsiung, Taiwan; 3 Department of Soil and Water Conservation, National PingTung University of Science & Technology, PingTung, Taiwan; 4 Research Center for Environmental Changes, Academia Sinica, Taipei, Taiwan; 5 Department of Geography, National Taiwan Normal University, Taipei, Taiwan; 6 Institute of Hydrobiology and Aquatic Ecosystem Management, University of Natural Resources and Life Sciences, Lunz, Austria; 7 WasserCluster Lunz (WCL), Inter-university Research Institute, Lunz am See, Austria; 8 Institute of Soil Research, University of Natural Resources and Life Sciences, Vienna, Austria; 9 Department of Life Science, Tunghai University, Taichung, Taiwan

## Abstract

Transport of riverine dissolved carbon (including DOC and DIC) is a crucial process linking terrestrial and aquatic C reservoirs, but has rarely been examined in subtropical small mountainous rivers (SMRs). This study monitored DOC and DIC concentrations on a biweekly basis during non-event flow periods and at 3 h intervals during two typhoon events in three SMRs in southwestern Taiwan between January 2014 and August 2016. Two models, HBV (the Hydrologiska Byråns Vattenbalansavdelning model) and a three-endmember mixing model, were applied to determine the quantities of DOC and DIC transport from different flow paths. The results show that the annual DOC and DIC fluxes were 2.7–4.8 and 48.4–54.3 t C km^−2^ yr^−1^, respectively, which were approx. 2 and 20 times higher than the global mean of 1.4 and 2.6 t C km^−2^ yr^−1^, respectively. The DIC / DOC ratio was 14.08, which is much higher than the mean of large rivers worldwide (1.86), and indicates the high rates of chemical weathering in this region. The two typhoons contributed 12%–14% of the annual streamflow in only 3 days (about 1.0% of the annual time), whereas 15.0%–23.5% and 9.2%–12.6% of the annual DOC and DIC flux, respectively, suggested that typhoons play a more important role in DOC transport than DIC transport. The end-member mixing model suggested that DOC and DIC export was mainly from surface runoff and deep groundwater, respectively. The unique patterns seen in Taiwan SMRs characterized by high dissolved carbon flux, high DIC / DOC ratio, and large transport by intense storms should be taken into consideration when estimating global carbon budgets.

## 1 Introduction

Transport of dissolved organic and inorganic carbon (DOC and DIC) by river systems is an important linkage among atmospheric, terrestrial, and oceanic C reservoirs (Meybeck and Vörösmarty, 1999; Battin et al., 2008). DIC derived from rock weathering is largely affected by tectonic activities, responsive to climatic change, and closely linked to atmospheric CO_2_ concentration over geological timescales (Lloret et al., 2011). By contrast, DOC mainly from the decomposition of particulate and dissolved organic matter (POM and DOM) is closely associated with different organic sources and physical environments (e.g., temperature, moisture). Both DOC and DIC availability in freshwater ecosystems controls dynamics of primary producers and microbial components in aquatic food webs (Maberly and Madssen, 2002; Maberly et al., 2015; Giesler et al., 2014). Globally, exoreic rivers can annually export 0.21 and 0.38 Pg C of DOC and DIC to the ocean (Huang et al., 2012). Although the quantity is small compared with the terrestrial C storage (about 2300 Pg C) (Battin et al., 2009; Cole et al., 2007; Ludwig et al., 1998), it has direct effects (i.e., combination of autotrophic or heterotrophic bacteria and CO_2_ emission) on downstream ecosystems (Lloret et al., 2013; Atkins et al., 2017). Large rivers yield approx. 1.4 and 2.6 t C km^−2^ yr^−1^ of DOC and DIC, representing 21.0% to 37.5% of the global riverine C export (Meybeck and Vörösmarty, 1999). Much of the variation in river export of DOC and DIC depends upon rock lithology, soil properties, climate, runoff, contact time (or flow velocity), aquatic primary production, UVB exposure, and streamwater pH (Wymore et al., 2017).

With the urgent demand for a precise global C budget and modeling, a thorough understanding of riverine C response to climatic and anthropogenic changes in different regions is needed (Meybeck and Vörösmarty, 1999). Among the global regions, humid tropical/subtropical regions are characterized by high biomass and rainfall export of large quantities of carbon (Galy et al., 2015; Hilton, 2017), with rivers between latitude 30° N and 30° S transporting 62% of the global DOC to the ocean (Dai et al., 2012). For these systems, rates of export (2.1 and 3.3 t C km^−2^ yr^−1^ of DOC and DIC, respectively) are much greater than the global averages (1.4 and 2.6 for DOC and DIC, respectively) (Huang et al., 2012). Thus, the tropical/subtropical regions are hypothesized as hotspots of DOC and DIC flux (Degens and Ittekkot, 1985; Lyons et al., 2002). However, studies on DOC and DIC transport in this region are rare.

For riverine DOC transport, the flush hypothesis argues that terrestrial C accumulates in the riparian zone and near-stream hillslopes in non-event flow periods and the accumulated C is subsequently flushed by major storms when the water table rises (Mei et al., 2014). Since DOC and DIC have different sources and different transport pathways that are active under different flow regimes, shifts in hydrologic flow paths would alter the quantity and ratio of DIC : DOC (Walvoord and Striegl, 2007). Understanding of shifts in the quantity and DIC : DOC ratio has become increasingly important because extreme climate events such as tropical cyclones are projected to become more frequent and intense as a result of global warming (Galy et al., 2015; Heimann and Reichstein, 2008). However, little is known about the processes and their underlying mechanisms of DOC and DIC export to rivers (Atkins et al., 2017). Specifically, the concentration and export of DOC and DIC are hypothesized as being different between regular and intense storm periods due to changes in the relative contribution from different flow paths, but studies to date provide little information on such shifts of DOC and DIC export.

In this study, we monitored DOC and DIC concentration during non-event flow periods (in biweekly frequency) and during two typhoon events (in 3 h intervals) for a subtropical small mountainous river in southwestern Taiwan. Based on the analysis of DOC, DIC, and major ions in combination with a hydrological model, HBV, and a three-endmember mixing model, we aimed at identifying different flow paths of DOC and DIC transport in different flow regimes. The specific objectives were to (1) compare the riverine DOC and DIC in concentration, flux, and ratio of DIC / DOC in three small mountainous rivers in Taiwan; (2) to understand the role of typhoon events in annual flux; and (3) to identify shifts in sources of DOC and DIC between non-event flow and typhoon periods.

## 2 Materials and methods

### 2.1 Study site

The study was conducted in Tsengwen River watershed, located in southwestern Taiwan. The Tsengwen River, which originates from Mt. Dongshui (2611 m a.s.l., above sea level), has a drainage area of 483 km^2^ with a mean terrain slope greater than 50%. The landscape is mainly covered by secondary forests dominated by *Eutrema japonica*, *Areca catechu*, and bamboo with small patches of betel nut and tea plantations. The annual mean temperature is approx. 19.8 °C with the lowest air temperature in January (17.8 °C) and the highest in July (21.1°C) (Central Weather Bureau, Taiwan, http://cwb.gov.tw, last access: 31 January 2017). The long-term mean annual rainfall is approx. 3700 mm yr^−1^, with approx. 80% occurring from May to October. Tropical cyclones, also known as typhoons in the western Pacific, with strong winds and torrential rainfalls, frequently strike the area and induce intensive mass movements (e.g., landslides and debris flows) within 2–3 days. These short-term, periodic, extreme events mobilize massive amounts of terrestrial materials to the ocean (Kao et al., 2010; Huang et al., 2017).

**Figure 1:**
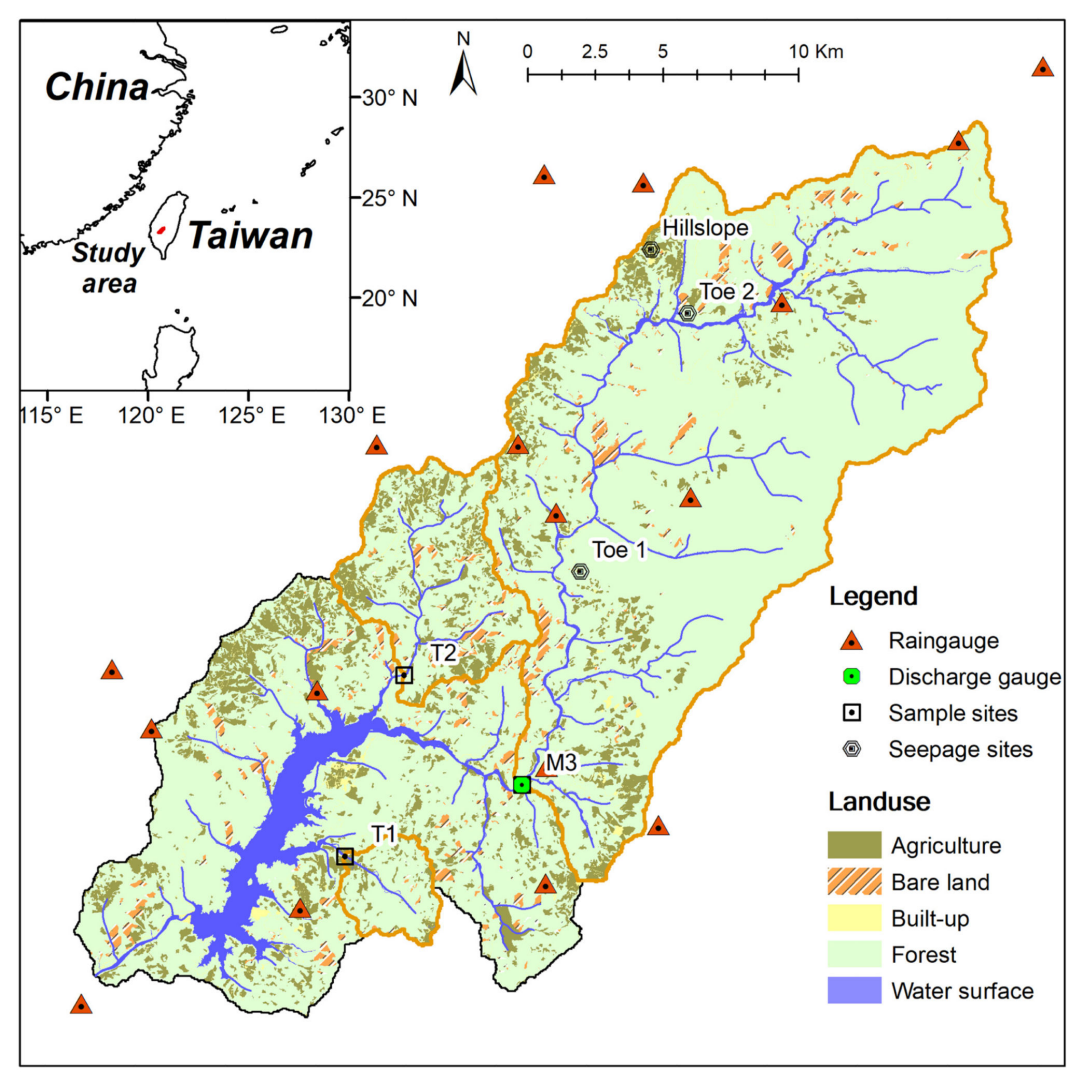
Location map of sampling sites, rain gauges and land cover pattern in Tsengwen catchment. The detailed descriptions of Hillslope, Toe 1 and Toe 2 are shown in Sect. S2.

Three sampling sites were set up: two at tributaries (T1, T2) and one at the mainstream (M3). The drainage areas for T1, T2 and M3 are 11.1, 40.1 and 274.1 km^2^, respectively (Fig. 1). There is a discharge station at M3 monitored by the WRA (Water Resources Agency, Taiwan, http://wra.gov.tw, last access: 31 January 2017) and 14 auto-recording precipitation stations maintained by the CWB (Central Weather Bureau, Taiwan). Land-use patterns were compiled from aerial photos, satellite images, and field surveys during 2004–2006 (National Land Surveying and Mapping Center, 2008). Forest is the main land use in the three catchments, accounting for 83.3%, 70.3%, and 87.7% for T1, T2, and M3, respectively. The proportion of agricultural land (i.e., betel nut and tea plantation) accounts for 14.0% and 23.0% of the area in catchments T1 and T2, but only 7.0% in catchment M3. Two other minor land uses are built-up areas and bare land. Built-up areas indicate buildings, farmhouses, and roads. Bare land includes the landslide scars, unplanted farms, or places under development/construction. The legacy of mass movement (i.e., landslide scars) induced by typhoons accounted for 3.0%–5.3% of the land area of the three catchments.

### 2.2 Sampling and chemical analysis

Streamwater was sampled biweekly between January 2014 and August 2016. Additionally, a high-frequency (2–3 h interval) sampling scheme was applied during two typhoon events (Typhoon Matmo, 21–23 July 2014, and Typhoon Soudelor, 6–8 August 2015). We took water samples from a bridge by lowering a set of four 1 L HDPE bottles (high-density polyethylene) into the river. A 1 L bottle of water (unfiltered) was used to measure water temperature, pH and electrical conductivity (EC) in the field. Another bottle of water sample was filtered (through pre-weighed and pre-combusted 0.7 μm GF/F filters) and stored at 4 °C in a refrigerator for further analyses of major cations and anions in the lab. Approx. 50 mL filtrate was acidified by H_3_PO_4_ for further measurement of DOC (Analytik Jena multi N/C^®^ 3100 Analyzer) with a detection limit of 4 μg L^−1^. Major anions $\left({\text{Cl}}^{-},{\text{NO}}_{3}^{-},{\text{SO}}_{4}^{2-}\right)$ were analyzed by ion chromatography (IC, Methrom^®^ 886 basic plus) with a detection limit of 0.02 mg L^−1^. Major cations (Na^+^, K^+^, Mg^2+^, Ca^2+^) were analyzed by ICP-OES (PerkinElmer Inc. – Optima 2100 DV) with a detection limit of 0.02 mg L^−1^. Note that the mean pH values were 8.75, 9.0 and 8.57 for sites T1, T2 and M3, respectively. In this kind of neutral and weak alkaline water body, ${\text{HCO}}_{3}^{-}$, which is the main component (over 90%) of DIC, can be estimated by the ion balance method. This method calculates the difference between the total dissolved anions $\left({\text{TZ}}^{-}={\text{Cl}}^{-}+2{\text{SO}}_{4}^{2-}+{\text{HCO}}_{3}^{-}+{\text{NO}}_{3}^{-},\;\text{in}\;\mu \text{eq}\;{\text{L}}^{-1}\right)$ and total dissolved cations TZ^+^ = Na^+^ + K^+^ + 2Ca^2+^ + 2Mg^2+^, in μeq L^−1^). The difference is attributed to ${\text{HCO}}_{3}^{-}$(Misra, 2012; Zhong et al., 2017). To affirm the estimated DIC through $\left[{\text{HCO}}_{3}^{-}\right]$, we also determined the DIC of some samples through the NDIR method (OI Analitical^®^ Aurora 1030W TOC). The strong relationship (*R*^2^ = 0.93) between calculated and measured DIC for the tested subset of non-event samples (*n* = 12) gives confidence in the accuracy of the values derived from the ion balance method.

### 2.3 Estimation of DOC and DIC flux

The daily concentration and fluxes of DOC and DIC were estimated by Load Estimator (LOADEST) using the following equation (Runkel et al., 2004):

(1) $$\text{ln(}\hat{F}\text{)=}a_{\text{0}}+a_{1}\ln(Q)+a_{2}\ln(Q^{2})+a_{3}\sin(2\pi \cdot \text{dtime})+a_{4}\cos(2\pi \cdot \text{dtime}),$$

where $\hat{F}$ indicates the estimated load (kg km^−2^ d^−1^); *Q* represents stream discharge (mm d^−1^) and “dtime” denotes the Julian day (in decimal form), respectively. In LOADEST, the inputs (*Q* and Julian day) were decentralized (observation minus average and then divided by the average) to avoid collinearity (Runkel et al., 2004). The coefficients, *a*_1_ and *a*_2_, are associated with *Q* representing the hydrological control. The other coefficients (*a*_3_, *a*_4_), which regulate the seasonal variation, can represent seasonal changes in the concentration and flux through optimization. The coefficients in Eq. (1) (*a*_0_, *a*_1_, *a*_2_, *a*_3_, *a*_4_) are estimated by the Adjusted Maximum Likelihood Estimation (AMLE, Cohn, 1988; Cohn et al., 1992) method built into the LOADEST program. Note that LOADEST was only used for the estimation of daily flux based on the biweekly sampling. The event-based fluxes were directly estimated by the flow-weighted method based on the high-frequency sampling. The event-based fluxes were converted into daily fluxes, thus updating the original daily fluxes. The indicators, NSE and *B*_p_, are used as performance measures. The NSE (Nash–Sutcliffe efficiency coefficient, Nash and Sutcliffe, 1970) calculates the explained variances and measures the performance as follows:

(2) $$\text{NSE}=1-\frac{\sum_{t=1}^{T}(Q_{\text{s,t}}-Q_{\text{o,t}}{)}^{2}}{\sum_{t=1}^{T}(Q_{\text{o,i}}-{\bar{Q}}_{\text{o}}{)}^{2}},$$

where the *Q*_o_ and *Q*_s_ indicate the observed and simulated streamflow (mm d^−1^) in time step *t*, respectively, and $\bar{Q_{\text{o}}}$ represents the average of the observed streamflow (mm d^−1^). The NSE ranges from negative infinity to 1.0. Zero and unity of NSE are equivalent to the expected value of the observations and a perfect match between estimations and observations. The *B*_p_ shows the yield bias in percent, defined as the estimations minus the observations over the observations.

(3) $$B_{\text{p}}=100\times \frac{\sum_{k=1}^{N}\left(\hat{F}-F\right)}{\sum_{k=1}^{N}F},$$

where *F* is the observed load, $\hat{F}$ is the estimated load, and *N* is the number of observations during the period.

### 2.4 Streamflow simulation

A conceptual hydrological model, HBV (the Hydrologiska Byråns Vattenbalansavdelning model, Parajka et al., 2013), was applied to simulate the daily streamflow and hourly streamflow of the two typhoon events for the M3 catchment. The details of the HBV model and streamflow simulation are described in Seibert and Vis (2012) and Sect. S1 in the Supplement. Briefly, HBV streamflow simulation uses rainfall, temperature, and evapotranspiration (estimated by temperature and humidity) to simulate the streamflow and its components (e.g., RSR: rapid surface runoff, SSR: subsurface runoff, and DG: deep groundwater). For daily streamflow simulation, the daily rainfall, temperature and relative humidity during 2002–2015 from 14 auto-recording weather stations of CWB were used in our simulations. The evapotranspiration was estimated by the Linacre method (Linacre, 1977) through the R package for evapotranspiration (Guo et al, 2016). The observed M3 streamflow was then used to adjust the parameters through the NSE. The calibrated parameter set of M3 was applied to T1 and T2 using their own climatic inputs to simulate their streamflow. For event simulations, a total of 13 events (during 2005–2015) in M3 were used to calibrate the event-based parameter set. We also affirmed the reliability of the event-based streamflow components derived from the HBV models using the EC, [Cl^−^], [Mg^2+^], and [Ca^2+^] through a three-endmember mixing model. All the details of the modeling work are presented in Sect. S1.

### 2.5 Endmember mixing analysis

Conceptually, the streamflow is composed of rapid surface runoff (RSR), subsurface runoff (SSR), and deep groundwater (DG) during rainstorms. DOC and DIC concentrations collected from streamwater were treated as a mixture from the three runoffs and the three-endmember mixing model was used to estimate their relative contributions. With the assumption of time-invariant sources (we discussed this in Sect. S2) and mass balance, the sources of DOC and DIC transported by the three flow paths can be represented by the following two equations:

(4) $$1={\left[Q\right]}_{\text{RSR},i}+{\left[Q\right]}_{\text{SSR},i}+{\left[Q\right]}_{\text{DG},i},$$

(5) $${\left[C\right]}_{\text{River},i}={\left[C\right]}_{\text{RSR}}{\left[Q\right]}_{\text{RSR},i}+{\left[C\right]}_{\text{SSR}}{\left[Q\right]}_{\text{SSR},i}+{\left[C\right]}_{\text{DG}}{\left[Q\right]}_{\text{DG},i},$$

where the footnotes of RSR, SSR, and DG present the rapid surface runoff, subsurface runoff and deep groundwater, respectively, and “*i*” indicates the time step. [*Q*] is the proportion of the corresponding runoff, with the sum of the three equal to 1 at any time step. The observed elemental concentration, [*C*]_River,*i*_ in the stream, is regarded as the mixing result among [*C*]_RSR_, [*C*]_SSR_, and [*C*]_DG_. Note that the streamflow and the quantities of the three components have been determined by the HBV model. Based on the known streamflow, runoff components and riverine DOC / DIC concentrations, the unknown endmembers can be estimated by comparing the observed and simulated riverine DOC / DIC concentrations. The details of the modeling procedure associated with (1) accuracy of streamflow components, (2) accuracy of the estimated C sources and (3) time-invariant assumption for endmembers are discussed in Sect. S2.

## 3 Results

### 3.1 Temporal dynamics of DOC and DIC concentration and flux

**Figure 2:**
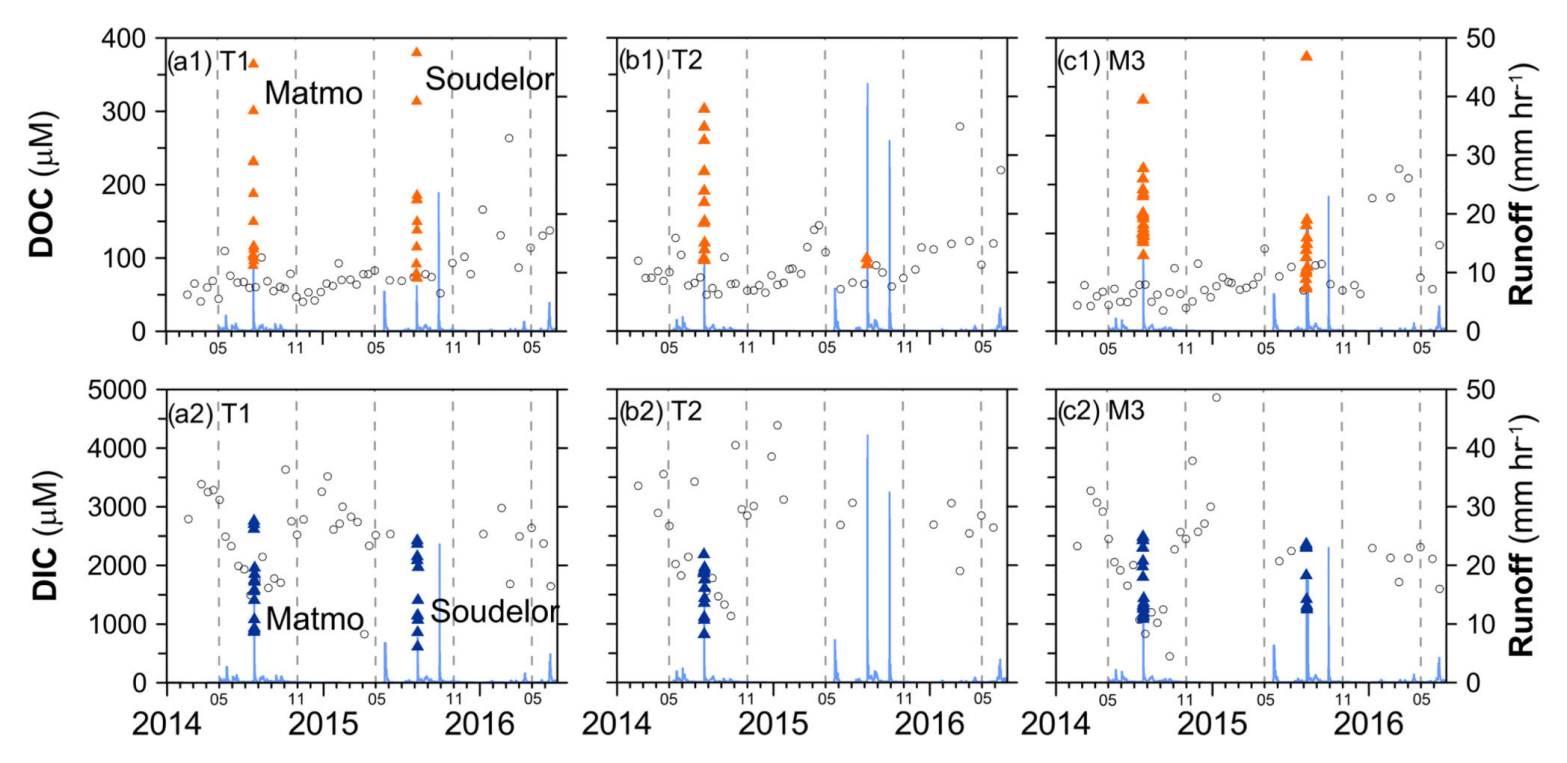
Observed DOC **(a1, b1, c1)** and DIC **(a2, b2, c2)** concentrations at the three sampling sites (left to right for sites T1, T2, and M3) during January 2014–August 2016. The blue line represents discharge. The black empty circles represent results of biweekly sampling and the orange and blue solid triangles indicate DOC and DIC concentrations during the typhoon events.

Most of the observed DOC concentrations of the three sites were less than 200 μM (or 2.4 mg C L^−1^) with no prominent seasonality, but rapid increases were observed during the two typhoon events (Fig. 2). The mean DOC concentration of the three sites varied from 48 μM in the dry season to 147 μM in the wet season (May–October), with an annual mean of 137 μM. In contrast, DIC concentrations varied widely from 1500 to 3500 μM during biweekly sampling of non-typhoon periods, illustrating a distinct seasonality. The DIC concentrations were higher in the dry season (November to the following April) and lower in the wet season, with a pronounced drop during typhoon events. The mean DIC concentration of the three sites varied from 2216 μM in the dry season to 1928 μM in the wet season, with an annual mean of 1951 μM (Table 2). Monthly fluxes of DOC and DIC were estimated satisfactorily by LOADEST, with *R*^2^ greater than 0.96, NSE of 0.88–098 and *B*_p_ of 0.4%–6.1% (Table 1). The acceptable performance in flux estimation supports the reliability of DOC and DIC fluxes from LOADEST. On the other hand, the performances of the estimated DOC and DIC concentrations by LOADEST were not as good as for flux. The *R*^2^ and NSE were 0.51–0.63 and 0.50–0.59 for DIC, slightly better than DOC, with *R*^2^ and NSE of 0.34–0.55 and 0.31–0.55, respectively.

**Figure 3:**
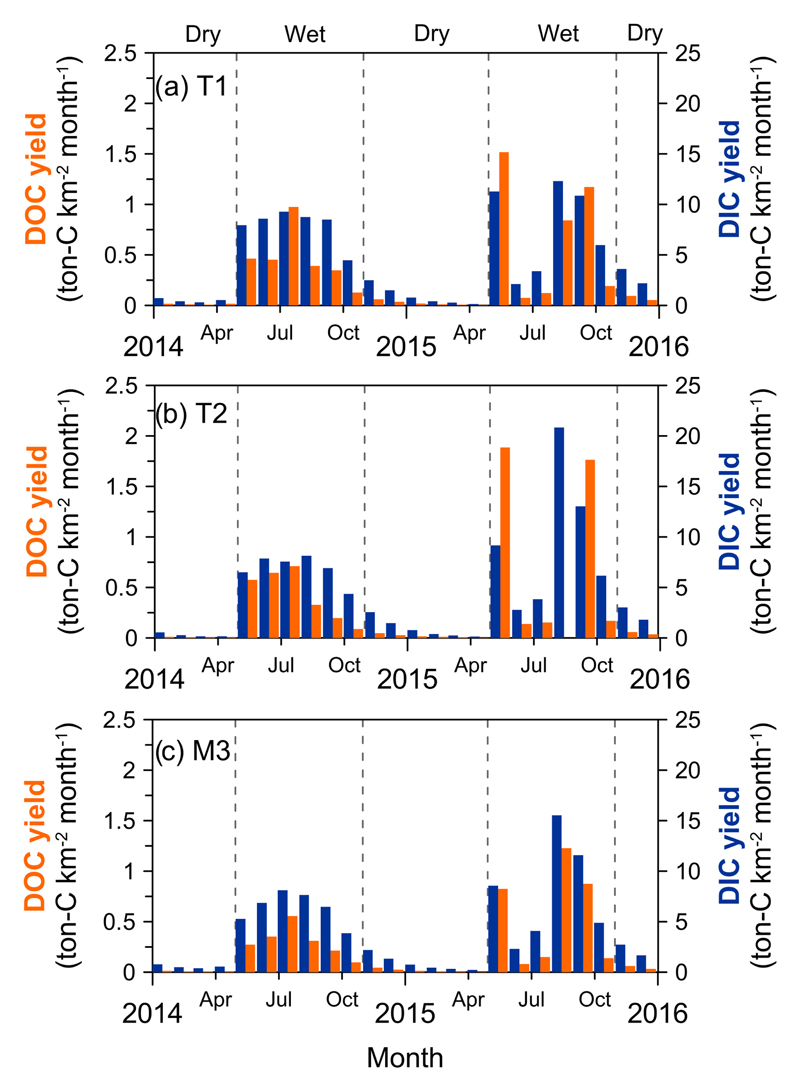
Monthly DOC and DIC yield (t Ckm^−2^ month^−1^) at the three sites, T1 **(a)**, T2 **(b)** and M3 **(c)**. Note that the typhoon event fluxes were taken into account.

The monthly DOC and DIC fluxes represented a distinct seasonal variation (Fig. 3). In general, the estimated DOC flux was 3.7 t C km^−2^ yr^−1^, with approx. 95% contributed during the wet season and the rest during the dry season, mostly due to higher discharge in the wet season. The annual DIC flux was approx. 52.1 t C km^−2^ yr^−1^, with approx. 88% occurring in the wet season and the rest in the dry season. A notable low export of DOC and DIC in June and July 2015 during the wet season was attributed to low rainfall, only 62 and 300 mm month^−1^ without typhoon invasions.

**Figure 4:**
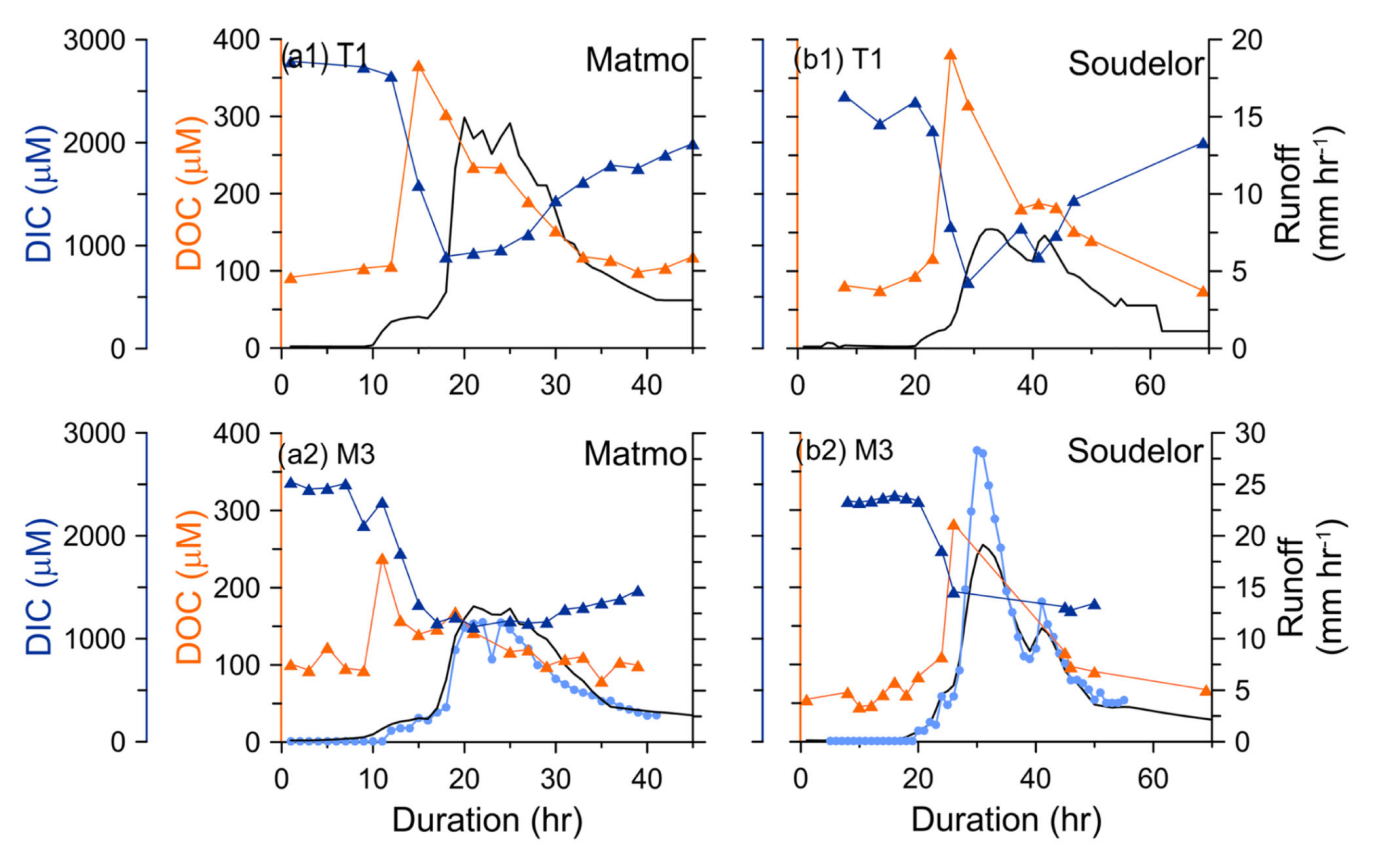
Temporal variation of DOC and DIC concentration during typhoon events. **(a)** is for Typhoon Matmo (22–24 July 2014) and **(b)** is for Typhoon Soudelor (7–10 August 2015). **(a1, b1)** and **(a2, b2)** are results of sites T1 and M3, respectively.

The variations of DOC and DIC concentrations of T1 and M3 during Matmo and Soudelor are shown in Fig. 4. The dataset of DOC and DIC at site T2, incomplete due to a road damage during Soudelor, is therefore not shown. During typhoon events, the DOC concentrations were about 100 μM in low-flow periods and they increased rapidly to more than 350 and around 270 μM for T1 and M3, respectively, just before the discharge peaks. After the discharge peaks, the DOC concentration quickly decreased to 100 μM, returning to levels prior to the typhoons. The DIC concentration showed an opposite temporal pattern. It was up to 2500 μM in low-flow periods; however, it gradually decreased with the increase in discharge during typhoon events to only 900 and 1200 μM in T1 and M2, respectively. During the recession period, the DIC concentration gradually increased to 2000 and 1500 μM for T1 and M3, respectively. The recovery of DIC concentration back to pre-typhoon levels was much slower than for DOC concentration.

### 3.2 Streamflow components and sources of DIC and DOC

After the calibration with eight historical events (occurring 2005–2013), the streamflow simulations of Matmo and Soudelor by HBV agreed well with the observed discharge as indicated by the high NSE values (0.82 and 0.89, respectively). In this modeling approach, rapid surface runoff (RSR) contributed approx. 40%–50% to the total flow, sub-surface runoff (SSR) accounted for approx. 25%, and the rest was attributed to deep groundwater (DG). The threeendmember mixing model and the ions (including Ca^2+^, Mg^2+^, Cl^−^, and EC) were used to evaluate the fractions of different runoffs which performed moderately well, with *R*^2^ values of 0.76, 0.73, 0.36, and 0.68 for Ca^2+^, Mg^2+^, Cl^−^, and EC, respectively (see Sect. S2 for details).

**Figure 5:**
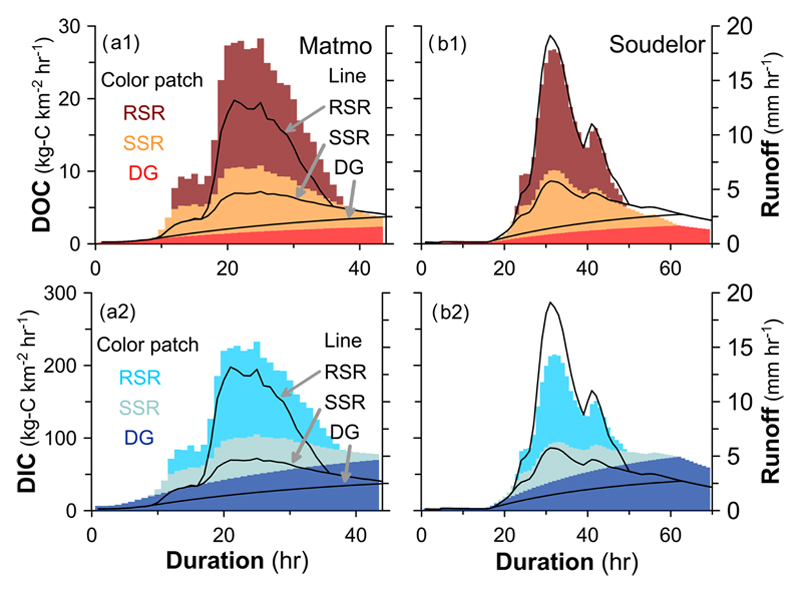
DOC and DIC from different sources during two typhoons at site M3. The colored patches present DOC and DIC flux from RSR (rapid surface runoff, upper patch), SSR (subsurface runoff, middle patch) and DG (deep groundwater, lower patch). The three stacked areas defined by black lines represent the hourly runoff from the three pathways (RSR, SSR and DG, from top to bottom, respectively).

Through the simple streamflow simulation and validation of its components, the proportions of runoff, DOC and DIC fluxes from the different flow paths were determined (Table 3), and the temporal variation of DOC and DIC fluxes transported via the flow paths is shown in Fig. 5. The two typhoon events, occurring only 1.0% of the year’s sampling period (i.e., 6 days), accounted for 12% and 14.0% of the annual discharge. DOC exported during Typhoon Matmo and Soudelor amounted to 382.5 kg C km^−2^ (or 15.0% of the annual flux) and 744 kg C km^−2^ (23.5%), respectively. Among the three flow paths, RSR was the main contributor, delivering approx. 40%–48% of DOC export during the typhoon periods, followed by SSR, about 37%, while DG only contributed about 20%. For DIC, the two events exported 3999.4 kg C km^−2^ (9.2% of the annual flux) and 6790.3 kg C km^−2^ (12.6%), respectively. The RSR, SSR, and DG transported approx. 29%, 21%, and 50% of DIC, respectively, during the two typhoon events. Since DG accounted for a low proportion of discharge, the high DIC flux from groundwater may be attributed to very high DIC concentrations. In sum, during typhoon events, the DOC was mainly transported by RSR due to the large amount of surface runoff flushing the large DOC pool stored at the land surface, whereas the DIC was mainly transported by DG owing to the very high DIC concentrations in the groundwater, even though the DG flow was small.

## 4 Discussion

### 4.1 Dissolved carbon dynamics in Taiwan SMR

Global mean DOC and DIC concentrations of large rivers are 479 and 858 μM, respectively, which is considerably larger than the means of 199 and 408 μM, respectively, for many SMRs around the world (Table 4). However, the global mean annual fluxes of DOC and DIC of large rivers are 1.4 and 2.6 t C km^−2^ yr^−1^, respectively, which is much lower than the means of 2.5 and 7.01 t C km^−2^ yr^−1^ for SMRs. For Oceania, which is characterized by high temperature, the mean DOC and DIC concentrations had been estimated at 399 and 1781 μM (Huang et al., 2012). On top of high rainfall, the fluxes of DOC and DIC in Oceania had been estimated at 8.0 and 34.0 t C km^−2^ yr^−1^, much higher than the global means of large rivers and SMRs. While the DOC concentrations in our study ranged around the means of global large rivers and SMRs, the DIC concentrations were much higher than the global means of both large rivers and SMRs (Table 4). The lower DOC concentrations but higher flux observed in our study and in the SMRs and Oceania islands suggest greater importance of streamflow for DOC export. On the other hand, the high DIC concentrations combined with high streamflow lead to the extremely high DIC export in Taiwan SMRs.

Globally, DOC flux is positively correlated with discharge and soil organic carbon (SOC) content, and negatively correlated with slope steepness (Ludwig et al., 1996a, b). Another study of global DOC flux indicated that the soil C : N ratio could be an important predictor for riverine DOC flux (Aitkenhead and McDowell, 2000). For SOC, Schomakers et al. (2017) reported that the SOC in shallow soils (< 100 cm) in Tsengwen watershed was only 2.9 ± 0.6 t C ha^−1^ 6 years after a landslide, and it increased to 75.7 ± 5.0 t C ha^−1^ after 41 years, being still lower than at an undisturbed reference site (117.9 ± 18.17 t C ha^−1^), which are lower values than reported for other SMRs (100–300 t C ha^−1^) (Scharlemann et al., 2014). The low SOC contents may not be the only cause of the observed low riverine DOC concentration in our study. The steep slopes, which result in restricted contact time between infiltrated water and the soils (Ludwig et al., 1996b; Hale and McDonnell, 2016), may additionally explain the low riverine DOC concentration in the studied SMRs. For aquatic ecosystems, steep landscape morphology, characterized by fast flows and short water residence times in the stream, limits an intense cycling of dissolved organic matter (DOM) in lotic ecosystems (Stutter et al., 2013). Although the high terrestrial productivity (owing to warm conditions) could consistently supply DOC to rivers, the high-flow velocities likely impair the productivity of lotic ecosystems. This could explain the low riverine concentrations in our study; however, due to abundant precipitation, the DOC fluxes were still higher than the global average.

Riverine DIC originating from rock weathering generally increases with increases in temperature, runoff and physical erosion rate (Maher and Chamberlain, 2014). Thus, the DIC concentration in SMRs gradually decreases from low to high latitudes (Table 4). In Oceania islands, the DIC concentrations are greater than 1000 μM, which is 2 times higher than the global average, most likely due to the large physical erosion and very high chemical weathering rates associated with the steep topography, high precipitation and high temperature (West, 2012). In our study, the DIC concentration and flux were 1951 μM and 52.1 t C km^−2^ yr^−1^. The DIC concentration was even as high as in the karst landscape (characterized by extraordinarily high DIC concentrations) of Wujiang (Zhong et al., 2017). In addition, high physical erosion rates, which expose fresh rocks, enhancing interaction with water, also provide conditions favorable for chemical weathering (Larsen et al., 2012, 2014; Lyons et al., 2005). The unique environmental setting likely causes the elevated DIC flux in our study, which is up to 10 times higher than the global mean of 2.6 t C km^−2^ yr^−1^ (Meybeck and Vörösmarty, 1999; Dessert et al., 2003).

The DIC / DOC ratios of the global large rivers, SMRs, and Oceania are 1.86, 2.80, and 4.25, respectively (Table 4). The DIC / DOC ratio can be used for improving the understanding of biogeochemical C processes such as photosynthesis and organic carbon mineralization in streams. DIC is the essential source of autotrophic photosynthesis and DOC of microbial decomposition (Lloret et al., 2011; Atkins et al., 2017). The global mean DIC / DOC ratio is around 1.86, indicating that DIC accounts for 65% of the total dissolved carbon in global large rivers. The DIC / DOC ratio in SMRs around the world is approx. 2.8, which could be due to (1) large DIC supply or limited DIC consumption, and (2) faster DOM decomposition. The DIC / DOC ratios in our catchments were 14.08, hence, much higher than those in other rivers of Oceania (4.25) and rarely seen at these ranges across the globe. From the viewpoint of a carbon mass balance, DIC could account for, at least, 90% of the total dissolved carbon export from the studied SMRs, which is a much higher share than that observed for global large rivers (approx. 65%). Therefore, when discussing global carbon dynamics, it should be kept in mind that the SMRs and Oceania islands, covering only a small fraction of the global land surface, probably have a disproportionately high flux of dissolved carbon to the ocean.

### 4.2 Sources of dissolved carbon in different flow regimes

**Figure 6:**
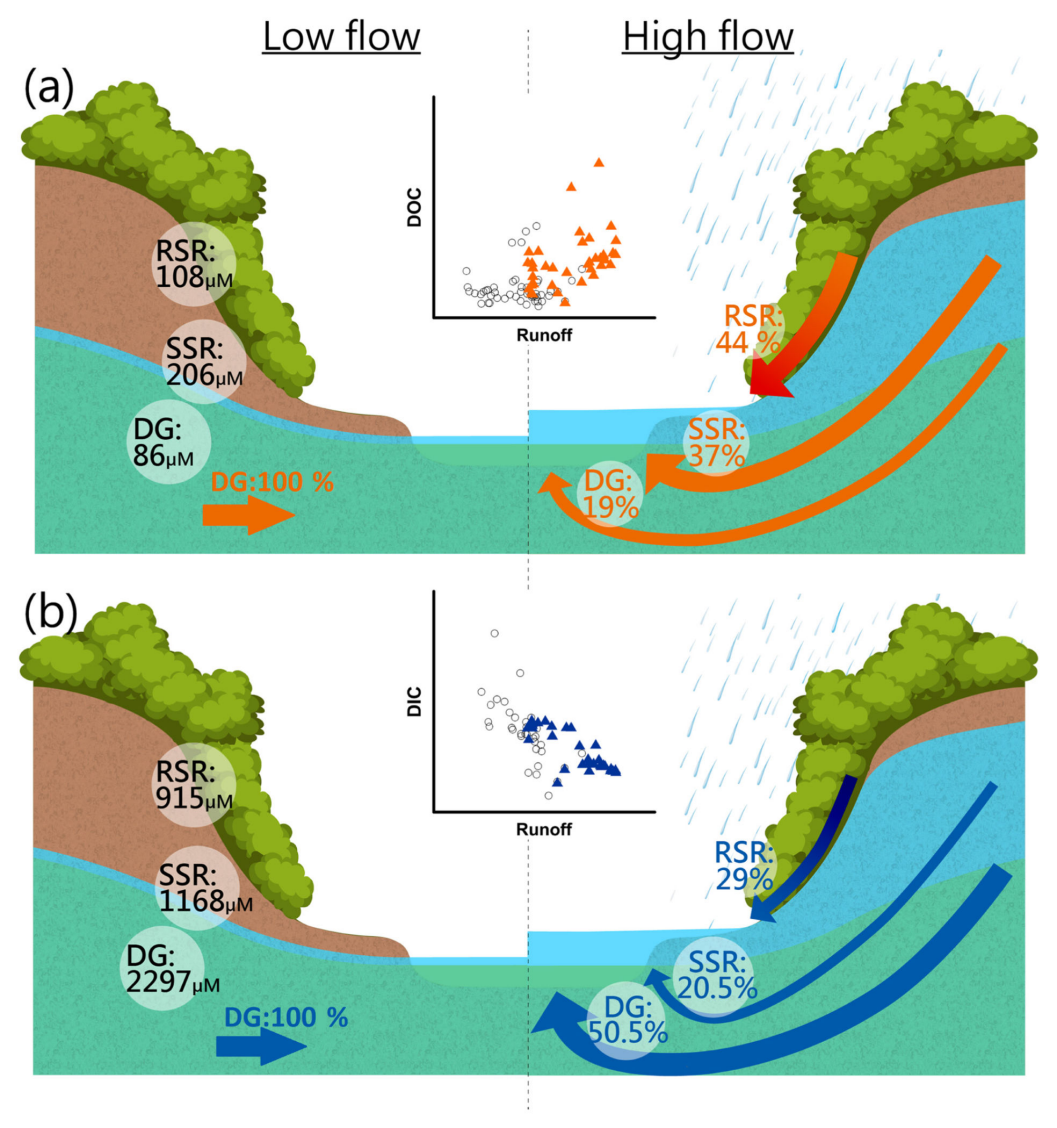
Conceptual model for **(a)** DOC and **(b)** DIC transport from different sources at low and high flows. The C–Q relations at low (black circle) and high (solid triangle) flows indicate that higher discharge would enhance DOC and dilute DIC concentrations. The estimated DOC and DIC concentrations from different runoffs are illustrated in the left part. The DOC and DIC concentrations at low flows are consistent with those from DG, since there is no other runoff at low-flow regimes. The arrows are in proportion to transport; RSR is the dominant flow path for DOC transport and DG for DIC at high flows.

The estimated DOC and DIC transport from different flow paths and the observed concentration–discharge (C–Q) relationships for DOC and DIC are illustrated in Fig. 6. In the C–Q relationship (the plots in the center of the figure), increasing streamflow enhances the DOC concentration but dilutes the DIC concentration, which confirms previous studies (e.g., Jin et al., 2014; Battin et al., 2003; Wymore et al., 2017; Zhong et al., 2017). The tighter C–Q relationship for DIC than for DOC indicates that the mechanisms of DOC transport cannot solely be explained by discharge control, possibly because microbial decomposition also played an important role (Yeh et al., 2018). Based on the source identification using the three-endmember mixing model, the DOC concentrations of the three sources (RSR, rapid surface runoff; SSR, subsurface runoff; and DG, deep groundwater) were estimated at 108, 206, and 86 μM, respectively. The estimated DOC concentrations in SSR and DG were only 1/3 to 1/2 of that in RSR. Thus, the land surface or the topsoils are likely the main source of DOC in our study. In fact, Schomakers et al. (2018) reported that the DOC concentrations in topsoils (0–10 cm) in the upstream area of M3 were 450 ± 33 μM under simulating typhoon conditions by ultrasonic treatments. It also suggests that RSR and SSR should be the main sources. On the other hand, the large discrepancy between our DOC concentration in RSR and that from ultrasonic treatments possibly indicates the dispersion of DOC from hillslope to stream. On the other hand, the lower DOC concentration in DG partly explains the low riverine DOC concentration in the low-flow period, since DG is the main contributor of baseflow. During high flows, RSR and SSR rapidly surge and flush terrestrial allochthonous DOC from soils into the stream, leading to the enhancement mode in the C–Q relationship, which is consistent with the flush hypothesis (Mei et al., 2014). On the other hand, the DIC concentration increased from 915 to 2297 μM with increasing depth of the flow path. The much higher DIC concentration in DG indicated that weathering likely takes place in the deep rocks (Calmels et al., 2011) and/or leaching of bicarbonate ions from the surface towards the subsoil and groundwater. Thus, the riverine DIC concentration gets strongly diluted by large contributions of RSR and SSR during high flows.

Two interesting questions arise from our study. First, what is the main DOC source in stream water during typhoon periods? Some studies suggested that the riparian zone is the main source of DOC during a rainstorm, as described by the flush hypothesis (Winterdahl et al., 2011; Wymore et al., 2017). However, hillslopes, as illustrated in our conceptual model, have also been proven an important source of DOC when rainstorms connect the hillslopes to streams by runoff (i.e., hydrological connectivity, Birkel et al., 2014). Further studies are suggested to clarify the relative importance of riparian zones vs. hillslopes for DOC export by using isotope techniques, for example, ^13^C of DOM and ^18^O of different runoff sources at different locations along hillslopes. Another interesting point is the change in the relative contributions of the three sources between non-event flow periods and extreme storm events in SMRs. For example, Lloret et al. (2011) argued that high water levels washed out the lower-molecular weight DOC from subsurface layers into streams. In our study, one typhoon could transport 12%–14% of annual streamflow, with 15%–23.5% and 9.2%–12.6% of annual DOC and DIC fluxes, which demonstrates the disproportional DOC and DIC transport by rainstorms. On average, three to six typhoons per year make landfall to Taiwan (Lin et al., 2017). Thus, the annual DOC and DIC flux contributed by typhoons may be as high as 50% and 30%, respectively. Lloret et al. (2013) reported that flash floods account for 60% of the annual DOC export and 25%–45% of the DIC export in small tropical volcanic islands, highlighting the important role of these extreme meteorological events. With projected global warming, the frequency and intensity of extreme rainfall are expected to increase, while mild rainfall tends to be reduced in Taiwan (Liu et al., 2009). Thus, streamflow may become more variable, scanter in the dry season, and higher in the wet season (Huang et al., 2014; Lee et al., 2015). In this regard, the water residence time would be longer in the dry season, which is very likely favorable for autotrophic production and, subsequently, DOC accumulation (Huntington et al., 2016). By contrast, the intensification of floods and the high-flow velocity would destroy the riverbed and reset the aquatic ecosystems. Under such conditions, the difference in the DIC / DOC ratio between dry and wet season would be exaggerated, with the potential for altering the biogeochemical C processes in aquatic ecosystems.

## 5 Conclusions

This study found that although the mean DOC concentrations in SMRs in southwestern Taiwan were as low as 99–174 μM, much lower than the global mean of 479 μM, the DOC flux was very high, 2.7–4.8 t C km^−2^ yr^−1^, 2–3 times the global average of 1.4 t C km^−2^ yr^−1^. The low DOC concentrations may be attributed to the steep landscape morphology, which limits the contact time of water with soils. On the other hand, the abundant rainfall still led to high DOC fluxes in the studied SMRs, revealing the importance of streamflow control for DOC export. By contrast, DIC concentration and flux are as high as 1805–2099 μM and 48.4–54.3 t C km^−2^ yr^−1^, much higher than the global mean of 858 μM and 2.6 t C km^−2^ yr^−1^. The very high DIC concentrations and fluxes likely result from active chemical weathering, and represent a large supply for aquatic photosynthesis. The mean DIC / DOC ratio of 1.86 for global large rivers indicates that the DOC accounts for 35% of the total dissolved carbon export. By contrast, the much higher DIC / DOC ratio (14.08) in our study indicates that DOC only accounts for 6.6% of the dissolved carbon, which might be unusual not only for Taiwan, but also for other SMRs.

The DOC and DIC fluxes during two typhoon events (occurring in only 1.0% of the annual time) contributed 15%–23% and 9.2%–12.6% of annual DOC and DIC flux, respectively, which highlight the role of extreme events in DOC and DIC transport. The enhancement of DOC during higher streamflow indicates the hillslope or riparian zone could be an important DOC source that was disproportionally flushed out during a high-flow regime. In contrast, the dilution effect of DIC associated with high streamflow implies that there was a large amount of runoff passing through sources with low DIC (e.g., land surface). The modeling demonstrated the patterns of DOC and DIC transport rapidly shifted during high- vs. low-flow regimes. The DOC was mainly from the land surface and flushed out by surface runoff, whereas the DIC was mainly transported by deep groundwater. However, the linkage of different C reservoirs to streams requires further investigations. Riparian zones and hillslopes have both been suggested as major DOC sources during rainstorms, but the exact sources and the DOC mobilization and transformation during different flow regimes in SMRs have not been comprehensively addressed. The high dissolved carbon flux, high DIC / DOC ratio, and large transport by rainstorms in SMRs should be considered in estimating global carbon budgets.

## Supplementary Material

Supplement

## Figures and Tables

**Table 1:** Performance metrics of estimated DOC and DIC flux at the three sites using LOADEST.

		Sample	Flux			Concentration	
	
	Site	number^1^	*R*^2^	*B*_p_^2^	NSE	*R*^2^	NSE
DOC	T1	76	0.98	4.1	0.93	0.53	0.41
	T2	64	0.98	1.3	0.97	0.55	0.55
	M3	85	0.96	6.1	0.88	0.34	0.31

DIC	T1	65	0.98	0.4	0.94	0.60	0.58
	T2	42	0.97	3.2	0.95	0.63	0.50
	M3	67	0.97	3.1	0.98	0.51	0.59

1 Sample number varied among catchments due to differences in site accessibility associated with road damage caused by typhoons or due to equipment failure.

2 *B*_p_ indicates flux bias in percentage, defined as the estimated minus observed values over the observed values.

**Table 2:** Concentrations and fluxes of DOC and DIC at the three sites during 2014–2015.

	DOC	DIC	DOC	DIC
	
Catchment	conc. (μM)		flux (t C km^−2^ period^−1^)	
Annual				
T1	138	2099	3.5	53.4
T2	174	1951	4.8	54.3
M3	99	1805	2.7	48.4
Average	137	1951	3.7	52.1

Wet season*				
T1	150	2097	3.3	46.7
T2	184	1890	4.7	48.6
M3	108	1798	2.5	42.6
Average	147	1928	3.5	45.9

Dry season				
T1	53	2113	0.2	6.7
T2	55	2672	0.1	5.8
M3	37	1863	0.1	5.9
Average	48	2216	0.1	6.1

* Wet and dry seasons are defined from May to October and from November to the following April in Taiwan.

**Table 3:** The fluxes of DOC and DIC, their contributions to annual fluxes (%) and the relative contributions (%) from three sources (rapid surface runoff, subsurface runoff and deep groundwater) at site M3 during the two typhoon events.

		*Q*_sim_	DOC	DIC
	
		(mm event^−1^)	(kg Ckm^−2^ event^−1^)	
Typhoon	Flux	248.4	382.5	3999.4
Matmo	Event/annual	12%	15.0%	9.2%
	Rapid surface runoff	40%	40%	24%
	Subsurface runoff	24%	37%	19%
	Deep groundwater	37%	23%	57%

Typhoon	Flux	328.0	744.5	6790.3
Soudelor	Event/annual	14%	23.5%	12.6%
	Rapid surface runoff	50%	48%	34%
	Subsurface runoff	25%	37%	22%
	Deep groundwater	25%	15%	44%

**Table 4:** The mean SMR annual concentrations and fluxes of DOC and DIC across the globe.

	Concentration (μM)		Flux (t km^−2^ yr^−1^)			
				
Region	DOC	DIC	DOC	DIC	DIC / DOC^1^	Reference
Global	479	858	1.44	2.58	1.86	Meybeck and Vörösmarty (1999)^2^

Small mountainous rivers^3^	199	408	2.5	7.01	2.80	
Subarctic streams	222	279	1.52	2.03	1.34	Giesler et al. (2014)
Temperate headwater	–	–	1.7	6.3	3.71	Argerich et al. (2016)
Tropical seasonal rainforest	308	500	1.02	2.43	2.38	Zhou et al. (2013)
Tropical volcanic islands^6^	75	513	2.5	19.6	6.60	Lloret et al. (2011)
Tropical volcanic islands^7^	215	339	5.7	4.8	1.39	Lloret et al. (2011)
Southwestern China (karst)	88	2,472	1.5	41.0	27.30	Zhong et al. (2017)

Oceania	399	1781	8.0	34.0^4^	4.25	Huang et al. (2012)
Papua New Guinea	321	1018	8.9	28.2	3.20	Alin et al. (2008)
Southeastern Australia subtropical rivers	360	1860	0.44	1.1^5^	10.71–13.38	Atkins et al. (2017)
Tseng-Wen River, Taiwan	137	1951	3.7	52.1	14.08	This study

1 DIC / DOC is calculated from either concentration or yield, depending on data availability.

2 The DOC and DIC concentrations were reversely calculated from fluxes; the details can be found in Huang et al. (2012).

3 The values were averages of the listed studies, but did not include Zhong et al. (2017), due to the specificity of karst landscapes.

4 The discharge (1572 mm yr^−1^) that we used is consistent with the GRDC dataset but about 10 times higher than the value reported by Huang et al. (2012).

5 The discharge during the sampling period was only one-third of the long-term average due to the ENSO effect.

6 and 7 indicate low- and high-flow conditions, respectively.
