# Supplementary material for: Dynamic responses of DOC and DIC transport to different flow regimes in a subtropical small mountainous river
Source: Hydrol Earth Syst Sci. Author manuscript; Available in PMC 2019 May 17. (PMC6522373; doi:10.5194/hess-22-6579-2018)
Supplement: Supplement [file EMS82720-supplement-Supplement.pdf]

Supplement of Hydrol. Earth Syst. Sci., 22, 6579–6590, 2018
https://doi.org/10.5194/hess-22-6579-2018-supplement
© Author(s) 2018. This work is distributed under
the Creative Commons Attribution 4.0 License.

Hydrology and Earth System Sciences
Open Access

Supplement of

Dynamic responses of DOC and DIC transport to different flow regimes in a subtropical small mountainous river

Yu-Ting Shih et al.

Correspondence to: (riverhuang@ntu.edu.tw)

The copyright of individual parts of the supplement might differ from the CC BY 4.0 License.

Supplementary Information I. Hydrological modeling for daily and hourly streamflow simulation

The HBV model (using TUWmodel, ver. 0.1-8., a R package) is a lumped rainfall–runoff model on a catchment scale with a series of three-layer connected storages. Each storage regulates its own runoff, namely, the rapid surface runoff (Q_{RSR}), slow surface runoff (Q_{SSR}) and groundwater (Q_{DG}). The simulated streamflow is the summation of the three runoff types at each time step. This model can be executed with a daily or hourly time step. Briefly speaking, this model considers the processes of evapotranspiration and the generation of the three runoff types. The actual evapotranspiration is proportional to potential evapotranspiration which is a function of temperature, solar radiation and wind speed. The factor of proportionality depends on the current state of the soil moisture content. As precipitation falls, it fills the first storage (upper soil layer). Once the rainfall exceeds the threshold of the upper soil layer, the rapid surface runoff (Q_{RSR}) is generated. On the other hand, the water in the upper soil layer follows a power law function of current soil moisture content to recharge into the lower soil layer. The recharge rate from the lower soil layer to groundwater is a constant. For each storage, the outflow follows the corresponding streamflow – storage relationship ($Q = S^k$) to generate its runoff. Finally, a transformation function (function of the parameters, B_{max} and C_{route}) which governs the channel routing is used to reshape the hydrograph at the catchment outlet. Parameter definition and the suggested ranges are listed in Table S1.

Table S1. Definitions and ranges of parameters in the HBV model.

Parameter	Unit	Description	Lower limit	Upper limit
LPrat	-	parameter related to the limit for potential evaporation	0	1
FC	mm	field capacity, i.e., max soil moisture storage	0	600
BETA	-	the non-linear parameter for runoff production	0	20
k_0	timestep^{-1}	storage coefficient for very fast response	0	2
k_1	timestep $^{-1}$	storage coefficient for fast response	2	30
k_2	timestep $^{-1}$	storage coefficient for slow response	30	250
L_{suz}	mm	threshold storage state, i.e., the very fast response start if exceeded	1	100
C_{perc}	mm day^{-1}	constant percolation rate	0	8
B_{max}	timestep $^{-1}$	maximum base at low flows	0	30
C_{route}	timestep^2 mm^{-1}	free scaling parameter	0	50

Daily streamflow simulation

The observed daily streamflow at M3 was used to calibrate the parameters through the performance measure of *NSE* and NSE_{log}. The R package DEoptim (Mullen et al., 2011) was applied to optimize the parameter set. The calculation of *NSE* has been described in the main text and NSE_{log} shares the same calculation method, but it uses the logarithmic form of streamflow for addressing the variation in low flow conditions. The calibration and validation periods were 2013-2016 and 2005-2012, respectively. The mean annual rainfall during calibration and validation were 2,979 and 3,666 mm yr^{-1} and the corresponding streamflows were 2,377 and 3,284 mm yr^{-1}. The simulations of the calibration were satisfactory with *NSE* and NSE_{log} of 0.83 and 0.81, respectively. Besides, a similar performance was found for the validation period with *NSE* and NSE_{log} of 0.79 and 0.81, respectively. The comparison between the observed and the simulated streamflow is illustrated in Fig. S1 and the calibrated parameter set is shown in Table S2. This calibrated parameter set at M3 was further applied to T1 and T2 using their individual climatic inputs to estimate the streamflow of T1 and T2.

Table S2. Calibrated parameter sets of the HBV model for daily simulation.

Parameter	Unit	Calibrated value
LPrat	-	0.94
FC	mm	285.19
BETA	-	2.13
k_0	day^{-1}	1.00
k_1	day^{-1}	2.02
k_2	day^{-1}	31.16
L_{suz}	mm	42.62
C_{perc}	mm day^{-1}	2.24
B_{max}	day^{-1}	8.83
C_{route}	day^2 mm^{-1}	39.46

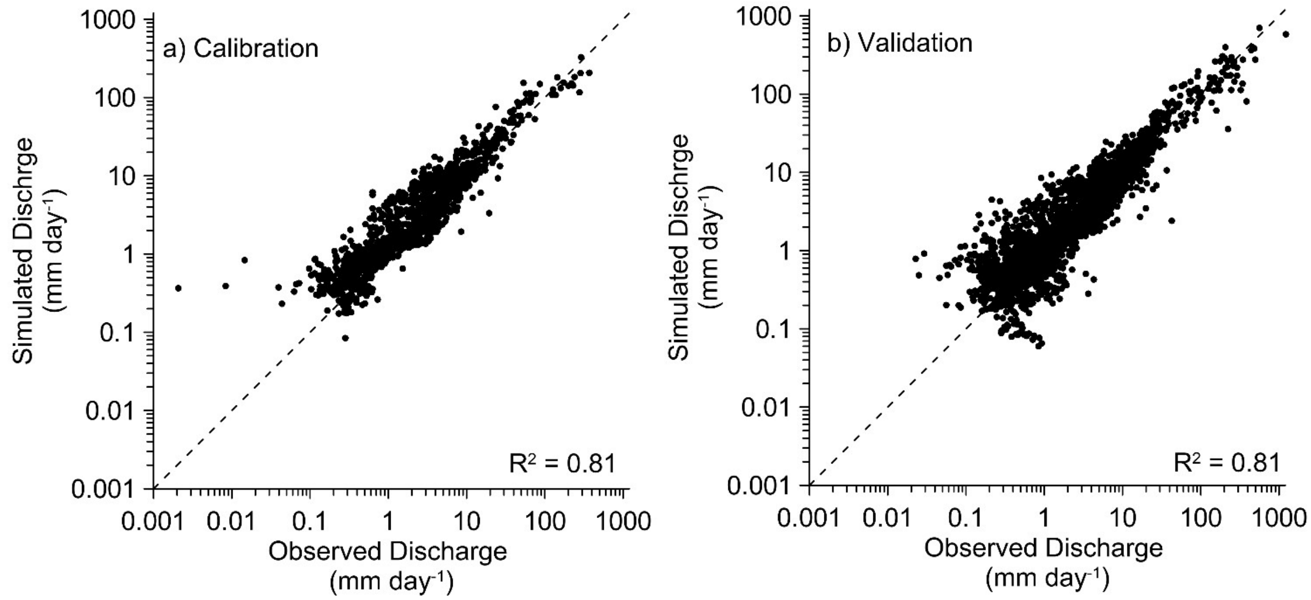

Figure S1. The comparison between observed and simulated daily streamflow during calibration (a) and validation (b) period. Dashed line indicates the 1:1 ratio.

Hourly streamflow simulation

The event simulation was carried out using daily simulation but with an hourly time step. The calibrated parameter set for the event simulation is shown in Table S3. The parameters of *FC*, *BETA*, and k_1 were quite different from those of the daily simulation indicating the hydrologic behaviors were distinctly different on daily and hourly timescale. The basic information and the simulation performances of the events were listed in Table S4. The average *NSE* and NSE_{log} for the calibration were 0.86 and 0.79, respectively. Most events could be satisfactorily modeled, except Typhoon Soulik. The total rainfall of Soulik was not the lowest, whereas the observed streamflow was the lowest probably due to the influence of rainfall heterogeneity. On the other hand, the average of *NSE* and NSE_{log} of the validation were 0.79 and 0.81, respectively, which was at a similar level as for the calibration. The comparison between the observed and the simulated streamflow is illustrated in Fig. S2.

Table S3. Calibrated parameter sets for hourly streamflow simulation.

Parameter	Unit	Calibrated value
LPrat	-	0.99
FC	mm	102.31
BETA	-	0.28
k_0	hr^{-1}	0.30
k_1	hr^{-1}	29.76
k_2	hr^{-1}	30.01
L_{suz}	mm	41.38

C_{perc}	mm hr^{-1}	3.41
B_{max}	hr^{-1}	2.87
C_{route}	hr^{2} mm^{-1}	28.79

Table S4. The modeling performances of hourly streamflow simulations

No.	Typhoon (Date)	Duration (hr)	Rainfall (mm)	Observed streamflow (mm)	Simulated streamflow (mm)	NSE	NSE_{log}
	**Calibration**						
1	Haitang (2005/07/17)	144	1157	1133	1041	0.92	0.61
2	Sepat (2005/08/18)	120	708	776	638	0.88	0.91
3	Sinlaku (2008/09/13)	75	836	758	709	0.95	0.93
4	Morakot (2009/08/06)	168	2205	2533	2103	0.89	0.74
5	Saola (2012/07/31)	84	470	356	362	0.97	0.94
6	Soulik (2013/07/13)	48	571	351	497	0.55	0.52
7	Trami (2013/08/21)	116	1025	853	810	0.90	0.77
8	Kong-Rey (2013/08/27)	147	357	478	421	0.86	0.93
	Average	113	916	905	823	0.86	0.79
	**Validation**						
9	Rainstorm (2012/06/10)	168	893	1066	849	0.57	0.68
10	Matmo (2014/07/22)	49	344	173	227	0.82	0.79
11	Soudelor (2015/08/05)	51	453	346	303	0.89	0.93
12	Dujuan (2015/09/24)*	115	592	621	494	0.88	0.89
13	Dujuan (2015/09/28)	53	359	371	266	0.81	0.77
	Average	87	528	515	428	0.79	0.81

*Dujuan had two distinct rainfall peaks and thus it was separated into two events.

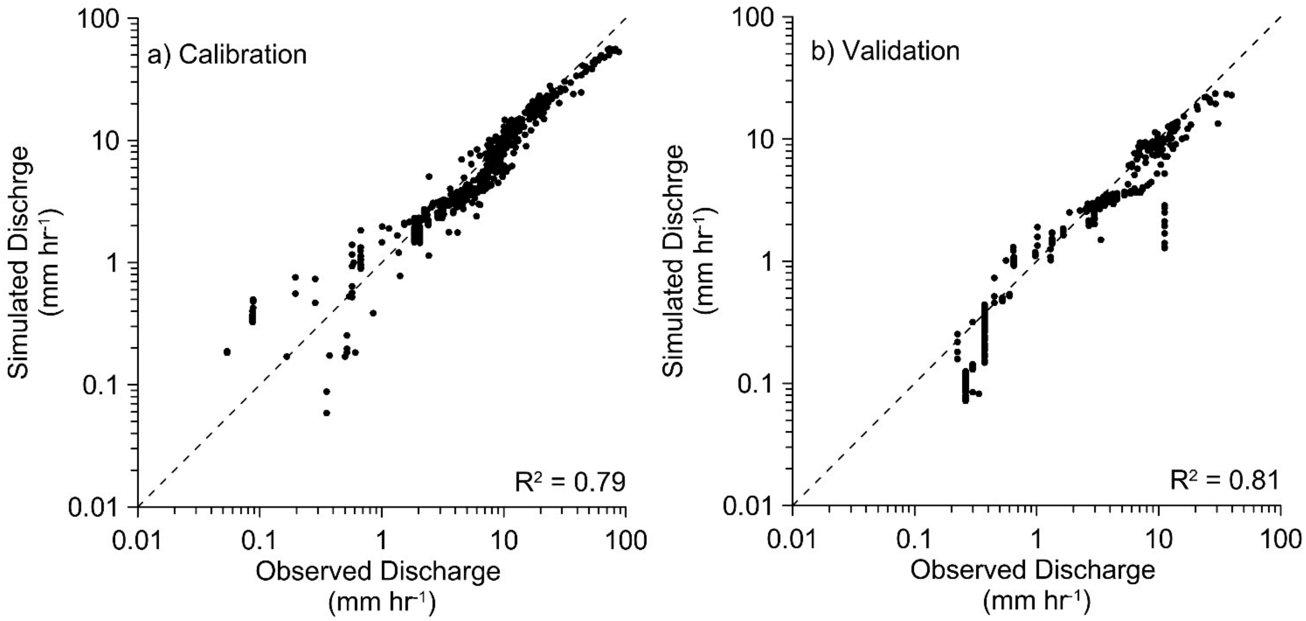

Figure S2. The comparison between observed and simulated streamflow during the calibration (a) and validation (b) events at an hourly scale. Dashed line indicates the 1:1 ratio.

Supplementary Information II. End-member mixing analysis for identification of C sources

End-member mixing models are widely used for identifying the mixing proportion and unknown sources of the mixture. In our case, Eq. 4 and Eq. 5 in the main text represent the mass balances of streamflow components and the DOC and DIC concentrations of the three sources. Theoretically, over 6 samples could be used to identify the end-members; however, the estimators should be constrained for avoiding biased inferences. Accordingly, three important issues should be addressed when using end-member mixing: (1) the accuracy of streamflow components (Q_{RSR}, Q_{SSR}, and Q_{DG}); (2) the accuracy of the estimation of end-members (e.g. C sources), and (3) whether the end-member is time-variant or time-invariant. Below, we described the validation of the three issues in our study.

Concerning accuracy of streamflow components, our hourly streamflow was satisfactorily simulated, which could support the estimations of the three streamflow components; however, the three components should be constrained or validated independently. Here, we introduced 4 chemical tracers (EC, Cl^-, Ca^{2+}, and Mg^{2+}) in streamwater to evaluate the accuracy of the three streamflow components. The R^2 values of the tracers for comparing the observed and the estimated of 3 end-members were 0.68, 0.36, 0.76 and 0.73 for EC, Cl^-, Ca^{2+}, and Mg^{2+}, respectively. The *NSE* values of the tracers were 0.27, 0.32, 0.76, and 0.55, respectively. Ca^{2+} and Mg^{2+} which are mainly derived from lithologic formations supported the estimated components. However, EC and Cl^-, which are easily altered by human disturbance and atmospheric deposition did not perform well. Despite the uncertainties in EC and Cl^-, the general promising agreement consolidates the reliability of the estimated three components.

Based on the above independent validation of the streamflow components, we identified the DOC and DIC end-members through the mixing analysis with fixed three runoff types. The estimated end-members of DOC were 108, 206, and 86 μM for Q_{RSR}, Q_{SSR}, Q_{DG}, respectively. The estimated DIC end-members were 915, 1168, and 2297μM for Q_{RSR}, Q_{SSR}, Q_{DG}, respectively. We also collected three water samples from seepage in hillslope as Q_{SSR} and drips in a tunnel as Q_{DG} (Table S5 and Fig. 1 in the main text). The promising agreement indicated that the C sources identification was reasonable, though the representativeness and spatial heterogeneity of sampling is still a challenge.

Table S5. The DOC and DIC concentrations from seepage and estimated sources

Samples*	Type	Observed DOC μM	Observed DIC μM	Estimated DOC μM	Estimated DIC μM
Hillslope	SSR	266	1033	206	1168
Toe #1	DG	124	2612	86	2297
Toe #2	DG	58	2726	86	2297

*: The three samples were collected in 2017-01-25, the driest period in our study catchment.

After constraining the streamflow components and the end-members of DOC and DIC, the time-variant assumption of end-members was also evaluated. Time-variant implies that the end-member should not be assumed constant and dynamic mixing behaviors should be considered. On the other hand, time-invariant end-members usually have slow turnover rates, indicating the end-member can release substance continuously (Mills et al., 2014). In practice, Bertrand-Krajewski et al. (1998) suggested to compare the cumulative substance export against cumulative runoff volume to examine whether the substance export is continuous. The continuous export implies a relatively slow turnover rate of the source. The cumulative DOC and DIC export against cumulative runoff volume are shown in Fig. S3. In this figure, both DOC and DIC export smoothly follow the 1:1 ratio of the cumulative export and runoff. The DOC release is a little higher than the runoff indicating that the DOC is likely enhanced or flushed out. Nevertheless, this plot suggests that the DOC and DIC are continuously being released during a typhoon event and a slow turnover rate could be inferred. Although we do not know whether the assumption of time-invariant end-member can hold for all unsampled cases in reality (a more robust methodology needs to be proposed), the continuous releasing and slow turnover rate likely support the time-invariant assumption in our two cases.

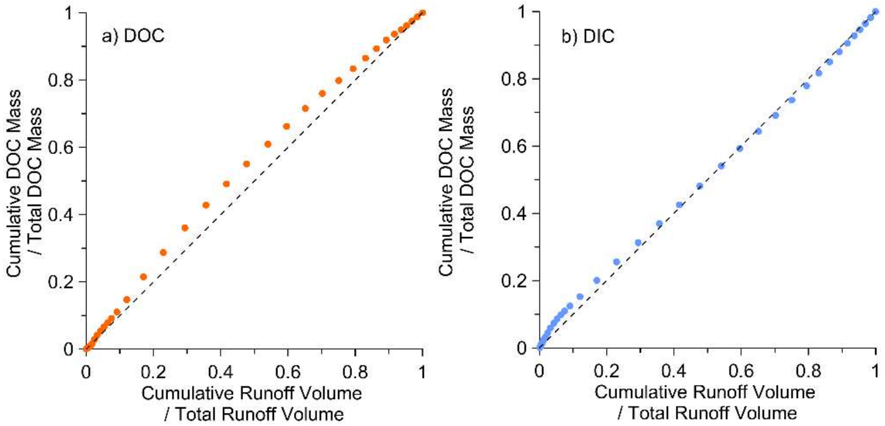

Figure S3. The cumulative export against cumulative runoff volume for (a) DOC and (b) DIC during typhoon Matmo. The dashed line shows the 1:1 ratio of cumulative export and runoff.

Supplementary Reference

Bertrand-Krajewski, J.-L., Chebbo, G., and Saget, A.: Distribution of pollutant mass vs volume in stormwater discharges and the first flush phenomenon, Water Res., 32, 2341-2356, https://doi.org/10.1016/S0043-1354 (97)00420-X, 1998.

Mills, R. T. E., Tipping, E., Bryant, C. L., and Emmett, B. A.: Long-term organic carbon turnover rates in natural and semi-natural topsoils, Biogeochemistry, 118, 257-272, 10.1007/s10533-013-9928-z, 2014.

Mullen, K. M., Ardia, D., Gil, D. L., Windover, D., and Cline, J.: DEoptim: An R Package for Global Optimization by Differential Evolution, Journal of Statistical Software, 40, 1-26, 2011.
